# Supplementary material for: Multi‐dataset identification of innovative feature genes and molecular mechanisms in keratoconus
Source: J Cell Mol Med. 2024 Sep 19;28(18):e70079. doi: 10.1111/jcmm.70079 (PMC11412914; doi:10.1111/jcmm.70079)
Supplement: Supplementary file 2 — Material S2. [file JCMM-28-e70079-s002.zip › 7.GSEA analysis/5.RNF217-AS1/KEGG_GLYCEROLIPID_METABOLISM.html]

Details for gene set KEGG\_GLYCEROLIPID\_METABOLISM[GSEA]

|  || Dataset | RNF217-AS1.group.cls#h\_versus\_l |
| Phenotype | group.cls#h\_versus\_l |
| Upregulated in class | h |
| GeneSet | KEGG\_GLYCEROLIPID\_METABOLISM |
| Enrichment Score (ES) | 0.2709335 |
| Normalized Enrichment Score (NES) | 0.93340534 |
| Nominal p-value | 0.5836502 |
| FDR q-value | 1.0 |
| FWER p-Value | 1.0 |
Table: GSEA Results Summary

  

Fig 1: Enrichment plot: KEGG\_GLYCEROLIPID\_METABOLISM      
 Profile of the Running ES Score & Positions of GeneSet Members on the Rank Ordered List

  

| SYMBOL | TITLE | RANK IN GENE LIST | RANK METRIC SCORE | RUNNING ES | CORE ENRICHMENT || 1 | GK2 | na | 1185 | 0.420 | 0.0214 | Yes |
| 2 | PNLIP | na | 1672 | 0.376 | 0.0571 | Yes |
| 3 | DGKH | na | 1684 | 0.375 | 0.1064 | Yes |
| 4 | MBOAT2 | na | 2431 | 0.320 | 0.1273 | Yes |
| 5 | AGPAT4 | na | 3421 | 0.274 | 0.1350 | Yes |
| 6 | LIPG | na | 4085 | 0.249 | 0.1489 | Yes |
| 7 | CEL | na | 4655 | 0.229 | 0.1627 | Yes |
| 8 | DGKB | na | 4824 | 0.223 | 0.1873 | Yes |
| 9 | PNLIPRP2 | na | 5504 | 0.199 | 0.1941 | Yes |
| 10 | DGKZ | na | 6049 | 0.182 | 0.2025 | Yes |
| 11 | GPAM | na | 6083 | 0.181 | 0.2254 | Yes |
| 12 | MGLL | na | 6459 | 0.169 | 0.2370 | Yes |
| 13 | GK | na | 6480 | 0.169 | 0.2587 | Yes |
| 14 | LIPF | na | 6786 | 0.159 | 0.2709 | Yes |
| 15 | PNPLA3 | na | 8623 | 0.114 | 0.2330 | No |
| 16 | DGAT2 | na | 9141 | 0.102 | 0.2317 | No |
| 17 | DGKD | na | 9445 | 0.096 | 0.2357 | No |
| 18 | LCLAT1 | na | 10363 | 0.080 | 0.2198 | No |
| 19 | AGPAT2 | na | 10737 | 0.074 | 0.2189 | No |
| 20 | AGK | na | 11511 | 0.063 | 0.2049 | No |
| 21 | GLYCTK | na | 12002 | 0.055 | 0.1980 | No |
| 22 | LPL | na | 13056 | 0.041 | 0.1732 | No |
| 23 | DGAT1 | na | 13693 | 0.034 | 0.1593 | No |
| 24 | MBOAT1 | na | 14213 | 0.028 | 0.1481 | No |
| 25 | PNLIPRP1 | na | 15412 | 0.015 | 0.1155 | No |
| 26 | AGPAT3 | na | 18541 | -0.016 | 0.0274 | No |
| 27 | GPAT2 | na | 19251 | -0.023 | 0.0100 | No |
| 28 | ALDH3A2 | na | 20121 | -0.031 | -0.0109 | No |
| 29 | DGKQ | na | 20482 | -0.035 | -0.0167 | No |
| 30 | AGPAT1 | na | 21058 | -0.040 | -0.0280 | No |
| 31 | AKR1A1 | na | 21302 | -0.043 | -0.0293 | No |
| 32 | GLA | na | 23202 | -0.064 | -0.0755 | No |
| 33 | ALDH9A1 | na | 23254 | -0.065 | -0.0685 | No |
| 34 | ALDH7A1 | na | 25259 | -0.095 | -0.1137 | No |
| 35 | DGKA | na | 27735 | -0.142 | -0.1663 | No |
| 36 | AKR1B1 | na | 28116 | -0.152 | -0.1572 | No |
| 37 | DGKG | na | 28176 | -0.153 | -0.1386 | No |
| 38 | ALDH1B1 | na | 28642 | -0.166 | -0.1300 | No |
| 39 | DGKE | na | 30971 | -0.241 | -0.1653 | No |
| 40 | LIPC | na | 33458 | -0.402 | -0.1839 | No |
| 41 | ALDH2 | na | 33631 | -0.423 | -0.1329 | No |
| 42 | AWAT2 | na | 34251 | -0.542 | -0.0790 | No |
| 43 | DGKI | na | 34571 | -0.701 | 0.0045 | No |
Table: GSEA details [plain text format]

  

Fig 2: KEGG\_GLYCEROLIPID\_METABOLISM      
 Blue-Pink O' Gram in the Space of the Analyzed GeneSet

  

Fig 3: KEGG\_GLYCEROLIPID\_METABOLISM: Random ES distribution      
 Gene set null distribution of ES for **KEGG\_GLYCEROLIPID\_METABOLISM**
